# Helminth Induced Suppression of Macrophage Activation Is Correlated with Inhibition of Calcium Channel Activity

**DOI:** 10.1371/journal.pone.0101023

**Published:** 2014-07-11

**Authors:** Arun Chauhan, Yuyang Sun, Biswaranjan Pani, Fredice Quenumzangbe, Jyotika Sharma, Brij B. Singh, Bibhuti B. Mishra

**Affiliations:** Department of Basic Sciences, School of Medicine & Health Sciences, The University of North Dakota, Grand Forks, North Dakota, United States of America; Louisiana State University, United States of America

## Abstract

Helminth parasites cause persistent infections in humans and yet many infected individuals are asymptomatic. Neurocysticercosis (NCC), a disease of the central nervous system (CNS) caused by the cestode *Taenia solium*, has a long asymptomatic phase correlated with an absence of brain inflammation. However, the mechanisms of immune suppression remain poorly understood. Here we report that murine NCC displays a lack of cell surface maturation markers in infiltrating myeloid cells. Furthermore, soluble parasite ligands (PL) failed to induce maturation of macrophages, and inhibited TLR-induced inflammatory cytokine production. Importantly, PL treatment abolished both LPS and thapsigargin-induced store operated Ca^2+^ entry (SOCE). Moreover, electrophysiological recordings demonstrated PL-mediated inhibition of LPS or Tg-induced currents that were TRPC1-dependent. Concomitantly STIM1-TRPC1 complex was also impaired that was essential for SOCE and sustained Ca^2+^ entry. Likewise loss of SOCE due to PL further inhibited NFkB activation. Overall, our results indicate that the negative regulation of agonist induced Ca^2+^ signaling pathway by parasite ligands may be a novel immune suppressive mechanism to block the initiation of the inflammatory response associated with helminth infections.

## Introduction

Helminth parasites infect over one third of the world's population and cause acute and chronic pathologies. They can also be remarkably efficient at establishing chronic infections, and yet many infected individuals remain asymptomatic [1], [2]. It has been proposed that helminth-induced regulation of host inflammatory response is essential for the parasite to evade the immune response and for establishment of long-term infections [2]–[4]. Concomitantly the down-regulation of host inflammatory response is beneficial to host survival as it prevents inflammation associated tissue pathology. For example in neurocysticercosis (NCC), which is the most common parasitic infection of the central nervous system (CNS), caused by the cestode *Taenia solium* (*T. solium*) [2], [5]–[9], viable cysticerci induce immune suppressive effects contributes to a long asymptomatic phase lasting 3–5 years [2]–[4]. In contrast, the loss of the parasite induced immune suppressive effects upon death of the parasite is thought to cause uncontrolled CNS inflammation resulting in tissue pathology and disease severity [2]–[4]. An understanding of the mechanisms underlying these effects can be clinically important as it may provide a foundation for therapeutics designed to either enhance inflammation when it is insufficient or attenuate it when it is excessive.

The host defense mechanisms for controlling microbial infections are broadly divided into two categories, innate and adaptive immune responses. A large number of studies on both humans and mice indicate that helminths trigger highly polarized adaptive immune response with increased numbers of CD4+ Th2 cells and regulatory T cells (Treg) [10]–[13]. The helminth-induced immune bias to a Th2 response activates and/or expands alternatively activated macrophages (AAMs) [12], [14]–[16] and generation of anti-inflammatory cytokines [11], [17]. AAMs function to promote wound healing and tissue remodeling [18]. Although much is known regarding modulation of adaptive immunity by helminths, our understanding of how these parasites regulate or even evade the innate immunity is very limited. Moreover, as initial host innate immune response is critical for the development of subsequent adaptive response, its regulation can be critical for helminth-induced immune suppression. In this regards, recent studies have demonstrated the critical role played by the Toll-like receptors (TLRs) family of proteins in host innate immunity [19]. Ligand recognition by TLRs has been linked to the manifestation of both infection induced inflammation or sterile- inflammation [20]. Indeed, recent studies have demonstrated that helminth molecules inhibit TLR ligand induced inflammatory cytokine production [17], [21]. However, the mechanisms involved in helminth induced inhibition of innate immune pathway activation still remains to be clearly understood.

Induction of inflammatory response by various stimuli has been shown to require increased cytoplasmic Ca^2+^ turnover for proper signal transduction [22]. In a cellular context, the onset of Ca^2+^ signaling is marked by increase in cytosolic Ca^2+^ both by release of Ca^2+^ from intracellular endoplasmic reticulum (ER) stores as well as influx across the plasma membrane (PM) [23]. This increase in intracellular Ca^2+^ triggers activation of downstream signaling pathways leading to inflammatory response [24]. The regulation of calcium signaling by parasites may be a novel immune suppressive mechanism to block the initiation of the inflammatory pathway. This is an unstudied area of research that warrants investigation.

In the present study using a murine model for NCC using the related cestode *Mesocestoides corti (M. corti)* [25]–[27], we observed a defect in myeloid cell activation/maturation in the helminth infected brains. Moreover, cestode soluble antigens inhibited TLR-ligand-induced pro-inflammatory cytokine production and NFκB activation *in vitro*. Additionally, exposure of PL (Parasite ligands from *M. corti*) inhibited not only TLR activation induced (LPS exposure), but also non-TLR agonist induced (thapsigargin exposure) activation of Ca^2+^ signaling pathway. The results indicate that the negative regulation of SOCE by cestode is a novel immune suppressive mechanism to dampen inflammation.

## Materials and Methods

### Ethics Statement

The animal usage protocols were approved by the Institutional Animal Care and Usage Committee at the University of North Dakota (protocol no. 1203–4 and 1109–3). All the procedures strictly followed the institutional and federal guidelines and all efforts were made to minimize animal suffering.

### Animals and antigens

Maintenance of the animals and tissue collection for performing experiments were conducted under the guidelines of the IACUC, the University of North Dakota system, the US Department of Agriculture, and the National Institutes of Health. Female BALB/c or C57Bl/6 mice used in this study were purchased from Charles River Laboratories, Wilmington, MA. *Mesocestoides corti* (*M. corti*) was kindly provided by Dr. Debrosky Herbert (Cincinnati Children's Research Foundation, Cincinnati, OH 45229, USA). *M. corti* metacestodes were maintained in peritoneal cavity of BALB/c mice by serial intra-peritoneal (i.p.) infection for propagation. Parasite ligands (PL) consisting of *M. corti* soluble factors was prepared from *M. corti* metacestodes by freezing and thawing. PL was passed through 0.2 µ filter for sterilization and protein content measured.

### Antibodies

To analyze myeloid cell maturation, Phycoerythrin (PE)-conjugated antibodies were purchased from BD PharMingen (San Diego, CA) include: M1/70 (anti-Mac1), 1D3 (anti-MHC-II), R35-95 (anti-CD80), B81-3 (anti-CD86) and HA4/8 (anti-OX40L). Biotinylated anti-mouse CD11b were also purchased from BD PharMingen. Biotinylated primary antibodies were detected using Alexa Fluor 488-labeled streptavidin (Molecular Probes, USA). For immunofluoresence (IF) staining, appropriate fluorescent conjugated secondary antibodies (Jackson Immuno Research Laboratories, West Grove, PA) were used.

### Murine NCC

Murine NCC was induced by intra-cranial (i.c.) injection (just below the dura) of 50 µl of HBSS containing approximately ∼40 *M. corti* metacestodes into 5 wk old C57BL/6 mice under short-term anesthesia [27], [28]. The anesthesia used was a 50 µl mixture of ketamine HCL and xylazine (30 mg/ml ketamine and 4 mg/ml xylazine) in phosphate buffered saline (PBS) that was injected intramuscularly as described previously [27]. Mock infected control mice were similarly injected with 50 µl sterile HBSS alone. At indicated times post-inoculation, anaesthetized animals were perfused through the left ventricle with 20 ml cold PBS, and brains were harvested to analyze expression of cell surface activation/maturation markers.

### Histology and immunofluorescence staining

The brains were immediately removed from perfused animals, embedded in O.C.T. resin (optimal cutting temperature), and snap frozen. Serial horizontal cryosections, 10 µm in thickness, were placed on silane prep slides (Sigma-Aldrich, St. Louis, MO). One in every five slides was fixed in formalin for 12 min at room temperature (RT) and stained with hematoxylin and eosin (H&E), as described previously [29]. The remainder of the slides were air-dried overnight and fixed in fresh acetone for 20 s at RT. Acetone-fixed sections were wrapped in aluminum foil and stored at −80°C or processed immediately for *in situ* IF microscopy analysis as previously described [26], [30]–[32].

### Effect on macrophage maturation and activation

Bone marrow was isolated from C57Bl/6 mice and the cells were differentiated to macrophages as previously described [33]. On day 6 of culture ∼90% cells were macrophages as determined by flow cytometry using macrophage specific markers CD11b and F4/80. The cells were plated at 1.5×10^6^ per well in 6-well plates and were incubated for 20 h at 37°C in medium alone, or in the presence of PL, LPS, or PL/LPS. BMDMs were harvested after 24 h and single cell suspensions were prepared at 2×10^7^ cells/ml in staining buffer (10% FCS in PBS) and pre-incubated with 1 µg of the 2.4G2 antibodies for 5–10 minutes on ice prior to staining. 50 µl of cell suspension (equal to 10^6^ cells) were dispensed into each tube or well along with a previously determined optimal concentration of cell surface specific antibody against MHCII, CD80, and CD86 in 50 µl of staining buffer. Cell surface expression of these maturation markers was measured on a BD LSR II flow cytometer (BD Biosciences).

To test the inhibitory effect of PL on secretion of pro-inflammatory cytokines, the cells were plated at 8×10^4^ cells per well in 96-well flat-bottom plates and were stimulated in medium alone, or in the presence of PL, TLR ligands alone (e.g. Pam3Cys4, dsRNA, LPS, ssRNA, or CpGDNA) or PL/TLR ligands as described above.Culture supernatants were collected 24 h after stimulation and measurement of TNF-α and IL-6 by ELISA according to the manufacturer's instructions (BD OptEIA, BD Biosciences).

### RNA isolation and Quantitative Real Time PCR (RT-PCR) analysis

Total RNA from BMDMs stimulated with medium alone, or in the presence of PL, LPS, or PL/LPS for 30 min, 2 h, 6 h and 24 h was isolated using Trizol reagent using manufacturers' instructions. One microgram of total RNA from each sample was reverse transcribed into cDNA by using a high capacity cDNA reverse transcription kit according to the manufacturers' instructions (Applied Biosystems, CA, USA). Transcript levels of IL-6 and TNF-αTLRs or the housekeeping ribosomal 18 S, were PCR amplified in each sample by using specific primers. Sequences of the specific primers used for 18 S, IL-6 and TNF-α are as follows: 18 S (sense) 5′- CAT GTG GTG TTG AGG AAA GCA-3′ and (antisense) 5′- GTC GTG GGT TCT GCA TGA TG-3′; IL-6 (sense) 5′- TTC ATC CAG TTG CCT TCT TG-3′ and (antisense) 5′- GGG AGT GGT ATC CTC TGT GAA GTC-3′; and (antisense) TNF-α (sense) 5′- ATC CGC GAC GTG GAA CTG -3′ and (antisense) 5′- ACC GCC TGG AGT TCT GGA A-3′. IL-6 and TNF-α mRNA levels were normalized to the mRNA level of the housekeeping gene 18 S in the same sample. The fold change was calculated by dividing the normalized value of the gene of interest in stimulated samples with the corresponding normalized value in unstimulated samples.

### Western blots

Stimulation experiments were set up as described before. BMDMs at 1.0×10^6^ per well in 12-well plate were pulsed with medium alone or PL for 30 min, followed by addition of LPS to a final concentration of 10 ng/ml or medium alone. A total of 1.5×10^6^ BMDMs was solubilized in 100 µl of 2× SDS PAGE sample buffer. Equivalent amounts of cellular extracts were resolved on 12% SDS-PAGE, and the separated proteins were transferred onto an Immobilon PVDF membrane (Biorad) for immunostaining. Blots were incubated with primary Ab against phosphorylated p38, ERK1/2, and JNK (Cell Signaling Technology, Beverly, MA), and bound Ab was visualized using secondary HRP-conjugated Ab and standard enhanced chemiluminescence (ECL, Amersham Biosciences).

### Calcium measurements

BMDMs or J774 macrophage cell line were plated at 1×10^6^ cells on collagen and poly-d-lysine coated 35 mm glass-bottomed culture dishes (MatTek, Ashland, MA) at 37°C. After 2–4 hr incubation, cells were washed twice with SES buffer (pH 7.4) containing 0.02% soybean trypsin inhibitor and 0.1% BSA and collagenase P (2.5 mg/8 ml of buffer) for 15–20 minutes at 37°C. Cells were incubated in SES buffer containing 2 µM Fura-2AM for 45–60 minutes at 37°C [34], [35]. Prior to performing Ca^2+^ measurements the culture dishes were washed with and placed in Ca^2+^-free SES buffer. Cells were stimulated with medium alone or with 25 µg/ml for 30 mins of PL before addition of Thapsigargin or LPS. Fluorescence measurements were performed by imaging the Fura-2AM-loaded macrophages using the Olympus IX50 microscope, with the excitation light provided by a Polychrome 4 (TILL Photonics) [34], [35]. Images were acquired using a Photometrics CoolSNAP HQ camera (Photometrics) and the MetaFluor software (Molecular Devices). Fluorescence traces shown represent [Ca^2+^]*_i_* values that are averages from at least 30–40 BMDMs and are a representative of results obtained in at least 3–4 individual experiments.

### Electrophysiology

All electrophysiological experiments were performed using previous protocol [34]–[36]. Coverslips with freshly isolated J774 cells were transferred to the recording chamber and perfused continually, through a custom-designed gravity-driven speed-controlled system at a rate of 5 ml/minute, with an external Ringer's solution of the following composition (mM): NaCl, 145; KCl, 5; MgCl_2_, 1; CaCl_2_, 1; HEPES, 10; glucose, 10; pH 7.4 (NaOH). The patch pipette had resistances between 3 and 5 mΩ after filling with the standard intracellular solution that contained the following (mM): cesium methane sulfonate, 145; NaCl, 8; MgCl_2_, 10; HEPES, 10; EGTA, 10; pH 7.2 (CsOH). Osmolarity for all solutions was adjusted with D-mannitol to 305±5 mmol/kg using a vapor pressure osmometer (Wescor). Patch clamp experiments were performed in the tight seal whole-cell configuration at room temperature (22–25°C) using an Axopatch 200B amplifier (Axon Instruments). Voltage ramps ranging from −90 to 90 mV over a period of 1 second were imposed every 4 seconds from a holding potential of 0 mV and digitized at a rate of 1 kHz. A liquid-junction potential of less than 8 mV was not corrected, and capacitative currents and series resistance were determined and minimized. For analysis, the first ramp was used for leak subtraction for the subsequent current records.

### Co-immunoprecipitation and western blotting

Co-immunoprecipitations and western analyses were carried out as described earlier [37], [38]. BMDMs were pulsed with medium alone or PL at 25 ug/ml for 20 min, followed by addition of 2 µm Tg, LPS or DMSO (0.1% v/v) for 5 min at 37°C, washed with ice-cold phosphate-buffered saline, and lysed in TNE buffer. Detergent-resistant LRD were isolated as above and resuspended in 1× radioimmune precipitation assay buffer supplemented with 0.1% SDS, 1% Triton X-100, 20% glycerol, 1 mm phenylmethylsulfonyl fluoride, and 1× protease and phosphatase inhibitor. Protein concentrations were adjusted to 1 mg/ml and immunoprecipitated with anti-STIM1. Immunocomplexes were separated using protein A plus-agarose beads (Pierce), eluted with 50 µl of 1× SDS dye and resolved in 4–12% NuPAGE gels (Invitrogen) followed by Western blotting as described previously using anti-TRPC1 antibodies [37], [38].

### Statistical analysis

We used the Student's *t*-test and one-way ANOVA for comparison of means of different groups [SIGMA PLOT 8.0 (Systat Software, San Jose, CA)]. A *P* value less than 0.05 was considered to be statistically significant.

## Results

### Myeloid cells display down-regulated maturation in murine NCC

**Figure 1:**
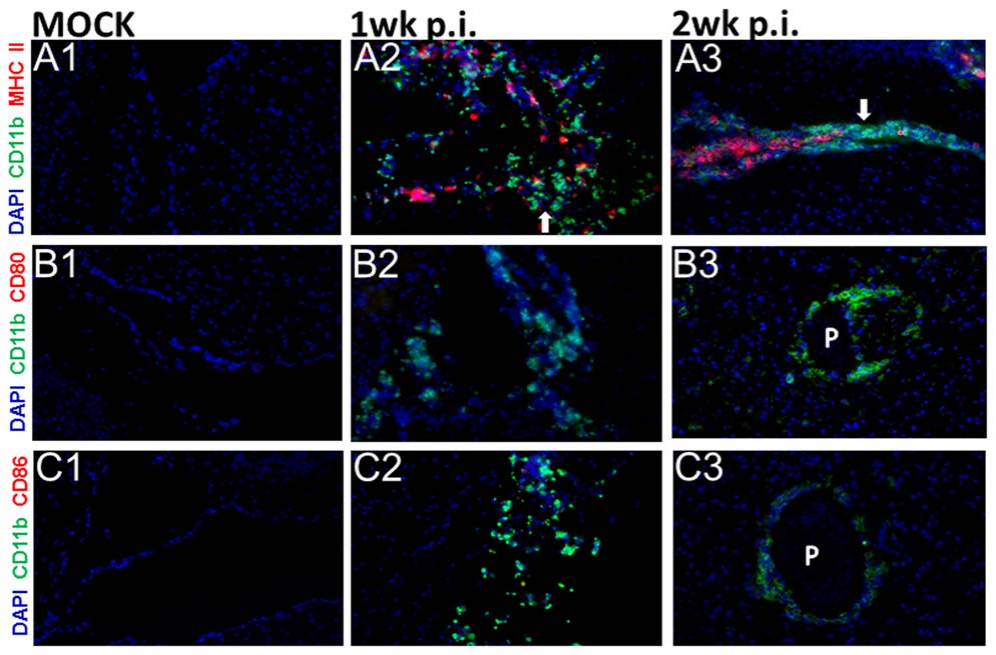
Infiltrating myeloid cells display reduced expression of surface maturation markers during murine NCC. *In-situ* IF staining was performed on frozen sections of HBSS infected (mock) and parasite infected brains of mice at 1 wk and 2 wk p.i. Brain cryosections were analyzed for expression of maturation markers MHC II, CD80, and CD86 using fluorochrome conjugated antibodies (red-PE) and macrophages specific marker CD11b (green-Alexa 488). Nuclear staining DAPI is depicted in blue. Staining for these maturation markers MHC-II (A1), CD80 (B1), and CD86 (C1) was undetected/barely detected in mock infected mice. Parasite infected mice brains displayed large number of infiltrating myeloid cells stained positive for CD11b (A2–A3, B2–B3, and C2–C3). MHC-II was undetected in many of the CD11b+ accumulated cells in the CNS of NCC mice at both 1 wk (A2) and 2 wk p.i. (A3) (arrow). CD80 (B2–B3) and Cd86 (C2–C3) were undetected/barely detected in the CD11b+ myeloid cells around the parasite (P) in the CNS of NCC mice both at 1 wk and 2 wk p.i. Results are from one representative experiment of atleast three independent experiments (n = 4 per each time point).

The expression and localization of myeloid cell surface maturation markers was examined by *in situ* IF microscopy in brain tissues from mock infected control and NCC mice. The kinetics of expression of MHC-II and the co-stimulatory molecules CD80, CD86, and OX40L were analyzed at 1wk, 2 wk, and 3 wk p.i., since upregulation of these molecules is considered an indicator of maturation of antigen presenting cells [39]. In mock infected animals, MHC-II, CD80, CD86, and OX40L positive cells were barely detected (Fig. 1A1, B1, C1, and data not shown). In murine NCC brains, a higher number of CD11b+ cells exhibited MHC-II staining as compared to the mock infected mice (Fig. 1A2, A3). However, many of these cells failed to display expression of MHC-II (Fig. 1A2, A3, arrow). Furthermore, in the parasite infected brains, expression of CD80 (Fig. 1B2, B3) and CD86 (Fig. 1C2, C3) and OX40L (data not shown) proteins were detected in few cells. Thus, a lack of detectable expression of MHC-II in many infiltrating myeloid cells, along with a low/diminished level of the costimulatory molecules, CD80, CD86 and OX40L, suggests a defect in the maturation of these cells in the CNS environment during helminth infection.

### PL inhibits LPS-induced activation of immature murine macrophages

**Figure 2:**
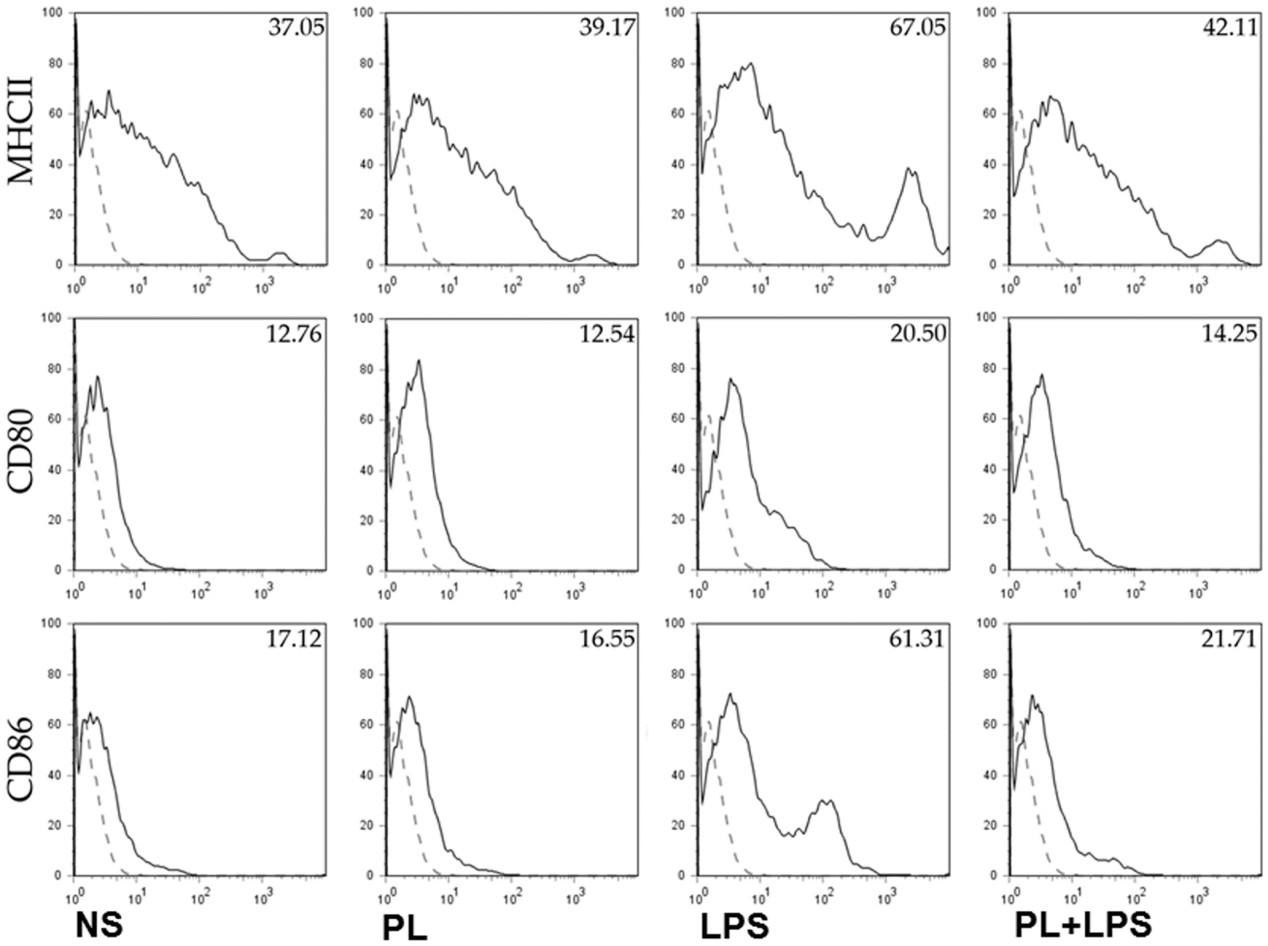
Helminth antigens downregulated surface maturation markers on BMDMs. BMDMs were pulsed with medium alone, PL at 25/ml, LPS at 10 ng/ml, or PL at 25 ug/ml twenty min prior to the addition of LPS to a final concentration of 10 ng/ml in the medium (PL+LPS) and cultured for a total of 20–24 h period. BMDMs were harvested and FACS analysis was performed to measure surface expression of MHC-II, CD80, and CD86. Dotted line in the histogram shows control staining with isotype/fluorochrome control Ab, and filled line shows signal with specific Ab.

Next the effect of helminth antigens on activation of BMDMs was tested in-vitro. Flow cytometric analysis was performed to measure cell surface expression of MHC-II and costimulatory molecules CD80, and CD86 in BMDMs exposed to various stimuli. BMDMs exposure to PL alone did not up-regulate MHC-II, CD80, and CD86 as measured by the median fluorescence intensity (MFI) (Fig. 2). As PL alone did not modulate expression of surface maturation markers, their immune suppressive effect on agonist induced activation/maturation of BMDMs was investigated. As expected, BMDMs exposed to LPS showed highly upregulated surface expression of MHC-II, CD80, and CD86 (Fig. 2). Interestingly, co-stimulation of BMDMs with PL and LPS resulted in inhibition of LPS induced upregulation of MHC-II and costimulatory molecules CD80, and CD86, (Fig. 2). Similar results were obtained for MHC-II, CD80 and CD86 in terms of percentage of BMDMs expressing them (data not shown). Together the results demonstrate that while PL alone does not elicit BMDM maturation, it efficiently inhibits LPS induced maturation of BMDMs.

### PL inhibits TLR ligand-induced inflammatory cytokine production in macrophages

**Figure 3:**
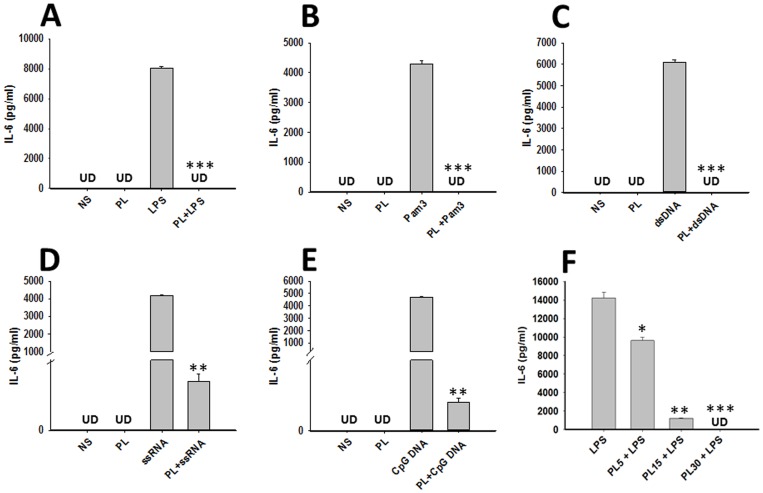
Effect of PL on cytokine production by BMDMs. BMDMs were pulsed with medium alone, PL at 25/ml, or various TLR ligands; LPS (10 ng/ml) (**A**), Pam3 Cys4 (Pam3) at 10 ng/ml (**B**), dsRNA (10 ng/ml) (**C**), ssRNA (10 ng/ml) (**D**), CpG DNA (1 µM) (**E**), or PL before the addition of respective TLR agonists in the medium. Cells were cultured for a total of 24 h period. The cytokine contents of IL-6 and TNF-α in culture supernatants were assayed by using specific sandwich enzyme-linked immunosorbent assays as recommended by the manufacturer (BD biosciences or R&D Systems). Cytokines detected below the assay detection limit for each stimulation are presented as UD (undetected). The mean ± SE concentration of cytokines in five independent experiments was determined. (F) BMDMs were pulsed with medium alone, PL at various concentrations; 5 ug/ml (PL5), 15 ug/ml (PL15) or 30 ug/ml (PL30), before the addition of LPS (10 ng/ml) in the medium. The cytokine contents of IL-6 in culture supernatants were assayed at 24 h as described above. Significant differences were measured by Student's *t* test and are denoted by asterisks (*, P<0.05, **, P<0.01 and ***, P<0.001).

Concomitant with the lack of upregulation of maturation markers, BMDMs exposed to PL alone failed to upregulate inflammatory cytokines IL-6 and TNF-α (Fig. 3, and data not shown). Thus, the effect of PL on agonist induced secretion of pro-inflammatory cytokines by BMDMs was tested. BMDMs were stimulated with various concentrations of PL, medium alone, TLR ligands Pam3Cys4 (TLR1/2 ligand), dsRNA (TLR3 ligand), LPS (TLR4 ligand), ssRNA (TLR7/TLR8 ligand), CpG DNA (TLR9 ligand) alone or in combination with PL. As expected, all TLR ligands alone activated BMDMs to secrete large amount of proinflammatory cytokine IL-6 (Fig. 3) and TNF-α (data not shown) in the culture supernatants. However, the TLR ligands induced secretion of these cytokines by BMDMs was completely inhibited by PL (Fig. 3A–E). This blocking effect PL was dependent on the concentration of PL and TLR ligands (Fig. 3F and data not shown) indicating the specificity of this effect.

**Figure 4:**
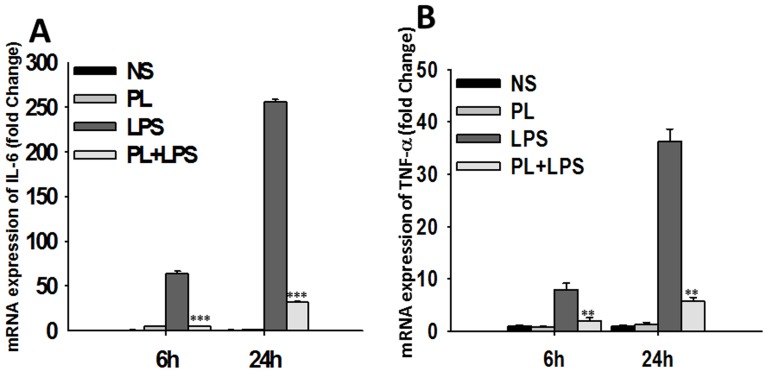
PL inhibits LPS-induced mRNA expression of IL-6 and TNF-α. BMDMs were pulsed with medium alone or PL, LPS, PL+LPS for 30 min, 2 h, 6 h, or 24 h. RNA was isolated and reversed transcribed to cDNA by using random primers. Levels of IL-6, TNF-α and housekeeping gene 18 S in these samples were measured by Real Time PCR analysis as described using SYBR green as the detection dye. IL-6 and TNF-α mRNA levels were normalized to the mRNA level of the housekeeping gene 18 S in the same sample. Fold change in mRNA expression of IL-6 (A) and TNF- α (B) after 6 h and 24 h stimulation over their respective baseline control level in unstimulated samples were expressed in arbitrary units. Data shown are representative of three independent experiments. Significant differences were measured by Student's *t* test and are denoted by asterisks (**, P<0.01 and ***, P<0.001).

In order to investigate if the blocking effect of PL was at transcription level, we next examined the effects of PL on LPS induced gene expression of IL-6 and TNF-α was analyzed. Upon stimulation, PL alone did not modulate gene expression of IL-6 or TNF-α at 30 min, 2 h, 6 h and 24 h post stimulation (Fig. 4, and data not shown). On the other hand, transcripts for IL-6 and TNF-α were elevated after LPS stimulation of BMDMs in comparison to unstimulated cells (Fig. 4, and data not shown). At 24 h post stimulation, a further increase in transcripts of IL-6 and in TNF-αat transcript level of both cytokines was observed upon LPS stimulation (Fig. 4). Costimulation of cells with PL and LPS together resulted in inhibition of LPS induced cytokine mRNAs by several fold, although both remained marginally elevated over non stimulated samples both at 6 h and 24 h (Fig. 4) as well as at 30 min and 2 h (data not shown). These results indicate that PL inhibits LPS- induced IL-6 and TNF-α production at transcript level.

### PL differentially inhibits LPS-induced NFκB and MAPK signaling activity

**Figure 5:**
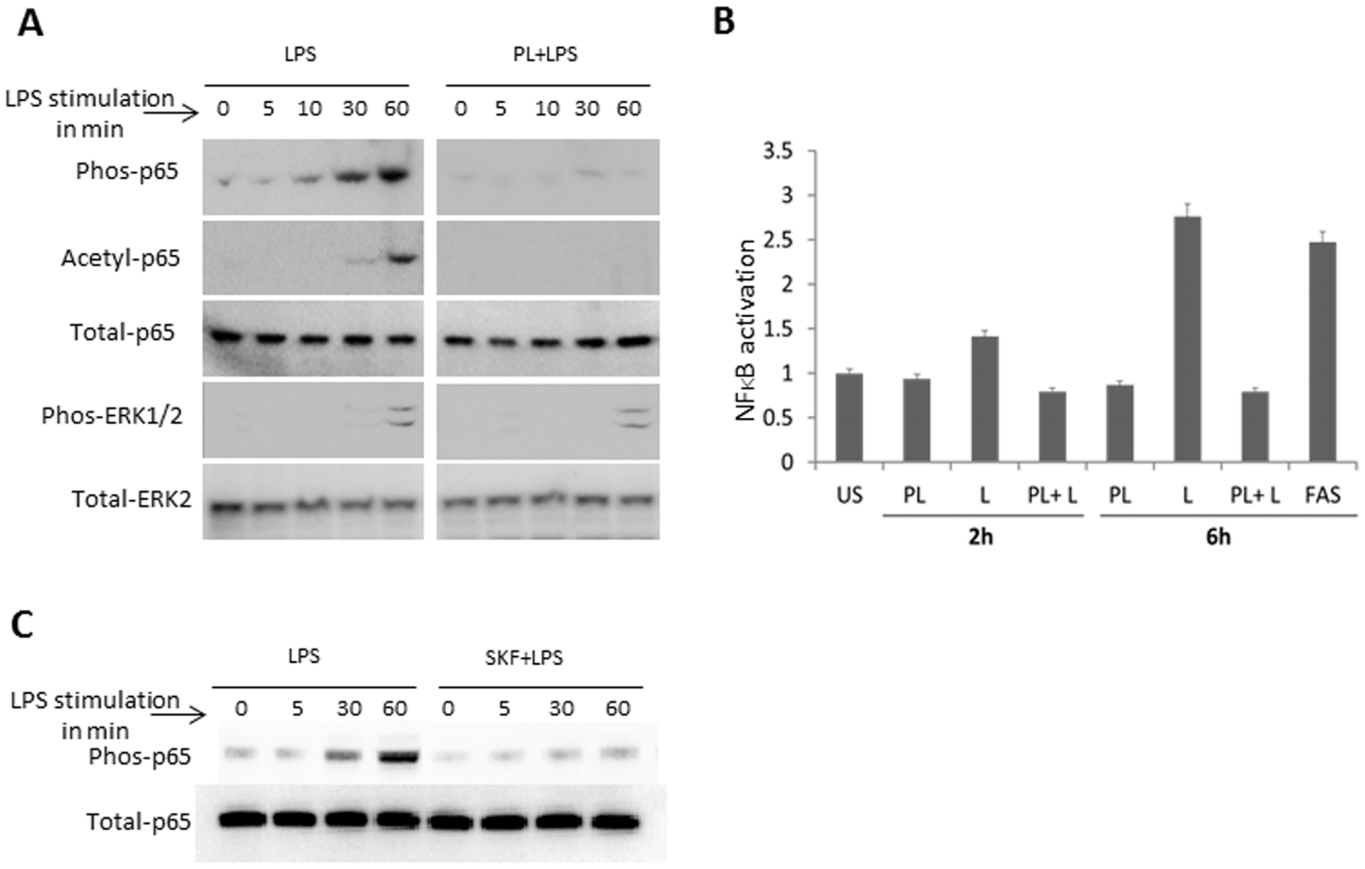
PL-mediated modulation in LPS-induced signaling. A) BMDMs were pulsed with medium alone or PL, LPS, PL+LPS. Extracts from BMDMs pulsed for mentioned period of time were electrophoretically separated, blotted and probed with Ab specific for phospho NFκB p65 (p65), p38, JNK, and ERK, or acetyl- NFκB p65, while anti- NFκB p65 anti-ERK as controls. Data shown here are representative of three independent experiments. B) Relative effects of PL, LPS, PL+LPS exposure on NFκB activation was measured in J774 macrophage cell line. FAS stimulation was used as a positive control. Data are normalized to luciferase activity in control J774 cells. Data indicates difference of values over their respective baseline control level in unstimulated (US) samples expressed in arbitrary units. C) BMDMs were pulsed with medium alone or LPS (10 ng/ml), SKF (10 µM) +LPS (10 ng/ml). Extracts from BMDMs pulsed for mentioned period of time were electrophoretically separated, blotted and probed with Ab specific for phospho NFκB p65 (p65), and ERK1/2, while anti- NFκB p65 anti-ERK as controls. Data shown here are representative of two independent experiments.

Interaction of microbial molecules with host receptors on APCs induces activation of downstream signaling cascades, invariably involving NFκB and MAPK pathway [40]. Thus the effect of PL on the activity of NFκB and MAPK such as p38, JNK and ERK1/2 in BMDMs was analyzed. Immunoblot analysis revealed that PL alone had no measurable effect on phosphorylation of p65, ERK1/2, p38, and JNK, as well as acetylation of p65, as compared to the untreated macrophages (data not shown). As expected, exposure of LPS induced an increase in phosphorylation and acetylation of NFκB p65 (Fig. 5A). Co-stimulation of PL and LPS substantially reduced LPS induced phosphorylation and acetylation of NFκB p65 (Fig. 5A). In contrast, PL failed to inhibit the LPS-induced increased phosphorylation of ERK1/2 (Fig. 5A), p38 and JNK (data not shown). Effect of PL was further examined by measuring NFκB-driven luciferase reporter. LPS exposure significantly increased the NFκB -driven luciferase reporter activity in J774 macrophages, which was decreased by PL exposure (Fig 5B). Taken together, our results showed that PL inhibited agonist-induced NFκB pathway activation, while it did not affect MAPK pathway activation by LPS.

### PL inhibits activation induced Ca^2+^ entry

**Figure 6:**
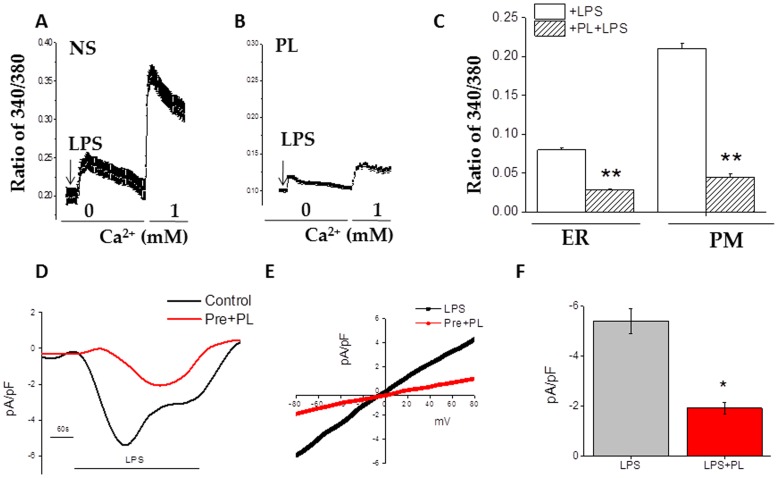
Helminth antigens inhibit LPS induced Ca^2+^ release and Ca^2+^ entry. Fura 2 fluorescence measurements in J774 cells. Cells were pulsed with medium alone, LPS at 10/ml, or pretreat PL at 25 ug/ml for 20 min before the addition of LPS to the medium. Analog plots of the fluorescence ratio (340/380) from an average of 0–50 cells are shown in (A) and (B). (C) The bar graph indicates the average data on calcium release (first peak) and calcium entry (second peak) under these conditions. ** indicate significance (p = <0.01). (D) Application LPS in bath solution induced inward currents at −80 mVin control and PL treated cells. Respectively IV curves under these conditions are shown in (E). Average (8–10 recordings current intensity at −80 mV are shown in (F). * indicate significance (p = <0.05).

Ca^2+^ entry through SOCE channels is essential for agonist induced NFκB activation and induction of inflammatory response. Indeed, co-exposure of LPS with SKF that inhibits the receptor-mediated influx of Ca^2+^ via voltage-gated calcium channels, abrogated LPS induced phosphorylation of NFκB p65 (Fig. 5C). To determine whether the helminth antigens affect the Ca^2+^ signaling, we performed Ca^2+^ imaging and electrophysiological experiments. Upon stimulation with PL alone in a Ca^2+^ free buffer, BMDMs or J774 macrophages did not show change in cytosolic Ca^2+^ level (Fig. 6B and D). However, as shown in Fig. 6A, stimulation of J774 cells with LPS in a Ca^2+^ free buffer showed an increase in cytosolic Ca^2+^ levels due to internal Ca^2+^ release (first peak, Fig. 6A), which corresponds to the store depletion (ER). Additionally, since ER store depletion induces SOCE, we measured Ca^2+^ influx, by the addition of 1 mM external Ca^2+^, which also showed a robust increase in cytosolic Ca^2+^ levels (second peak, Fig. 6A). But, exposure of cells with PL reduced LPS-stimulated cytosolic Ca^2+^ levels (Fig. 6B). This inhibition of SOCE by treatment with PL was statistically significant (Fig. 6C). Similar results were obtained when BMDMs were used (data not shown). Furthermore, electrophysiological recordings on J774 cells were performed to identify the SOCE channel involved. As shown in Fig. 6D and E, addition of LPS initiated a non- selective Ca^2+^ currents, which was inhibited in cells that were pretreated with the PL. Additionally, the IV properties of the channel were similar as observed in TRPC1-dependent Isoc (store-operated currents) [41] in J774 macrophage cell line (Fig. 6E, F), which were again inhibited by the addition of PL. These results further suggest that PL inhibits TRPC1-mediated Isoc.

**Figure 7:**
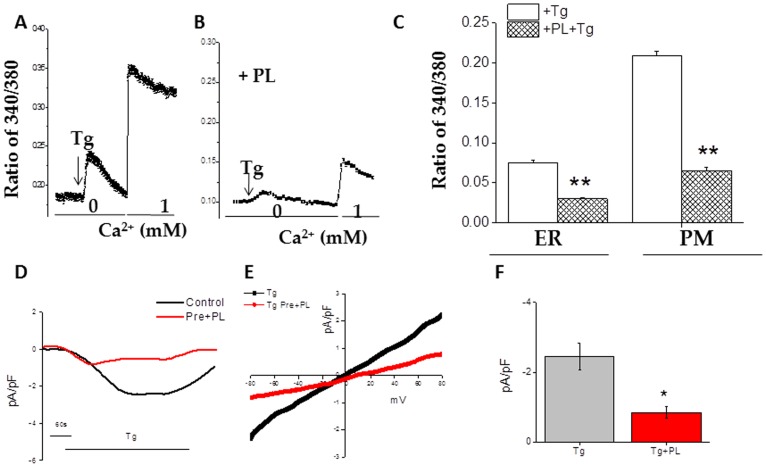
Helminth antigens inhibit Tg induced Ca^2+^ release and Ca^2+^ entry. Fura 2 fluorescence measurements in J774 cells. Cells were pulsed with medium alone, Tg at 1/ml for 20 mins before the addition of LPS to the medium. Analog plots of the fluorescence ratio (340/380) from an average of 0- 50 cells are shown in (A) and (B). (C) The bar graph indicates the average data on calcium release (first peak) and calcium entry (second peak) under these conditions. ** indicate significance (p = <0.01). (D) Inward currents were induced upon store depletion at −80 mV in control and PL treated cells Respectively IV curves under these conditions are shown in (E). Average (8–10 recordings current intensity at −80 mV are shown in (F). * indicate significance (p = <0.05).

The suppressive effects on non-PRR pathway dependent Ca^2+^ influx was also examined in Tg treated J774 cells or BMDMs with or without PL (Fig. 7). As expected, exposure of Tg increased Ca^2+^ release from ER (1^st^ peak) in J774 cells (Fig. 7A). Moreover, Tg induced store-depletion that activate plasma membrane Ca^2+^ channels allowing an influx of Ca^2+^ into the cytosol (Fig. 7A, C) was also decreased in cells exposed to PL (Fig. 7B, C). Similar results were also obtained when BMDMs were used (data not shown). Moreover, electrophysiological recordings were performed to identify the SOCE channel(s) involved. As shown in Fig. 7D, store-depletion initiated a non- selective Ca^2+^ current, which was similar as observed with LPS. The IV relationship showed a non-selective currents that reversed at 0 mV (Fig. 7E, F), which is similar as observed in TRPC1-dependent Isoc (store-operated currents) in other cells [41], and was significantly inhibited by the addition of PL, without changing the properties of the channel. Together, these data suggest that PL inhibits both PRR and non-PRR activated Ca^2+^ influx in macrophages, which is mediated via TRPC1.

**Figure 8:**
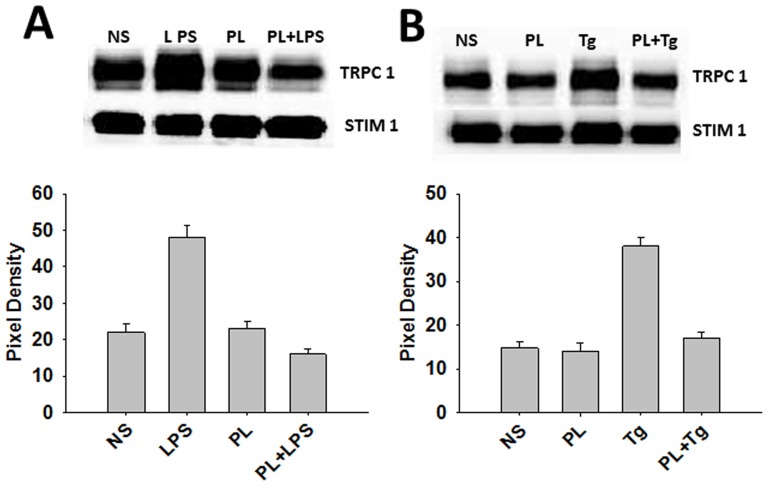
Helminth antigens impairment of TRPC1-STIM1 channel assembly. Co-immunoprecipitation of TRPC1 and STIM1 from BMDMs in resting, LPS-, and Tg-stimulations. A) Cells were pulsed with medium alone, PL at 25 ug/ml, LPS at 10 ng/ml, or PL before the addition of LPS to the medium. B) Cells were pulsed with medium alone, PL at 25 ug/ml, Tg at 2 µM, or PL before the addition of Tg to the medium. Immunoprecipitation of TRPC1-STIM1 complex from cell was prepared by using Anti-STIM1 Ab. Anti-TRPC1 was used for western blot detection in both Fig. 8 A and B. The average pixel intensity of the respective bands from three independent experiments was measured. This was done using the imaging software Quality One-4.6.7 (Bio-Rad).

SOCE is activated by redistribution of STIM1 into puncta in discrete ER-plasma membrane junctional regions where it interacts with and activates store-operated channels such as TRPC1 [37]. In order to determine whether the PL stimulation of cells affects the interaction between STIM1 and TRPC1 on PM, immunoprecipitation were performed using STIM1 antibodies to pull down TRPC1 in proteins isolated from J774 cells stimulated with LPS or Tg with or without PL, and assessed for the presence of TRPC1 by western blot (Fig. 8). As compared to control unstimulated cells, stimulation with PL for 30 min in Ca^2+^-free medium resulted in no change in TRPC1-STIM1 association. A substantial increase in the STIM1 interaction with TRPC1 was observed upon stimulation LPS or Tg alone. However, when cells were exposed to PL, there was a relative decrease in the LPS- or Tg- induced STIM1 interaction with TRPC1 (Fig. 8). Together these data demonstrate that store depletion by LPS and Tg induces recruitment of STIM1 and TRPC1 to PM and the STIM1 and TRPC1 interaction was inhibited by helminth antigens.

## Discussion

Helminth parasitic diseases, including NCC are chronic infections in which the organisms persist in host over long periods of time [42]. The persistent infections can cause acute and chronic pathologies presenting a major health care burden worldwide, particularly in developing countries. In contrast, as helminths are master regulators of host inflammatory response [43], [44], it has been proposed that the rise in autoimmune diseases in the developed world could be a direct result of the successful complete elimination of helminth parasites in these communities [41], [45], [46]. This warrants an improved understanding of helminth induced immune suppressive/anti-inflammatory responses, including in NCC. Here we report: 1) a diminished expression of maturation markers in myeloid cell in CNS microenvironment during murine NCC; 2) the cestode soluble antigens inhibit TLR ligand induced inflammatory mediator production in macrophages; 3) we show, for the first time, helminth antigens inhibit both PRR and non-PRR activated Ca^2+^ influx in macrophages; and finally, 4) helminth antigen through interaction with TRPC1 that inhibits TRPC1-STIM1 interaction, thereby inhibiting SOCE signaling pathways, which may be an important mechanism associated with helminth induced inhibition of innate immune pathway activation.

Inflammation is a complex process which is interplay of various host determinants. One of the important steps of agonist induced inflammation has been shown to require Ca^2+^ for activation of downstream signaling pathways. In a cellular context the onset of Ca^2+^ signaling is marked by increase in cytosolic Ca^2+^ both by release of Ca^2+^ from intracellular ER stores as well as influx across the PM [23]. This increase in intracellular Ca^2+^ triggers activation of downstream signaling pathways leading to agonist induced inflammatory response [47]. Results presented here clearly demonstrate that although, helminth antigens themselves do not modulate basal level of cytosolic Ca^2+^, they abolished the inflammatory stimuli induced increase in intracellular Ca^2+^ accumulation by blocking Ca^2+^ release and SOCE. We also showed that helminth antigens inhibit agonist induced TRPC1 activation on the PM of J774 macrophages. In this regard, we and others have previously reported PM as the major site of regulation of TRPC1-SOCE [37], [48]–[53]. Upon agonist induced activation, STIM1 is recruited into specific cellular domains where the ER and PM are juxtaposed [54], [55]. Moreover, our previous studies have shown that TRPC1 channel clusters with STIM1 after Ca2+-store depletion in the ER-PM junctional regions [37]. Importantly, this molecular rearrangement involving TRPC1 (i.e. store depletion induces association of TRPC1-STIM1) is involved in the activation of TRPC1-SOCE. The results from this study demonstrate lower levels of LPS induced inflammatory responses in macrophages that are treated with *M. corti* PL, which strongly correlates with the reduction in TRPC1-SOCE. Moreover, the data presented herein show that agonist induced clustering of STIM1 with TRPC1 was down-regulated by PL. Importantly, PL treated cells also displayed reduction in LPS induced NFκB activation. This is supported by our earlier studies demonstrating dependence of NFκB activation on TRPC1-mediated SOCE that is induced by TRPC1-STIM1 interactions [56]. Thus, taken together the results from the present study strongly support the possibility of a novel immune suppressive mechanism employed by helminths to hijack Ca^2+^ signaling complex in order to inhibit development of host inflammation.

Recently helminth molecules have been shown to down regulate innate immune pathway activation, particularly TLR4 ligation induced inflammatory response. Among them, FhHDM-1 from *Fasciola hepatica* has been shown to bind LPS and in that process thought to prevent recognition of LPS by TLR4 and LPS induced production of inaflammatory mediators *in vivo* and *in vitro* [57]. Other helminth immunomodulatory factors Lacto-N-fucopentaose III [12], omega-1 [58], [59], and sm16 [60] from *schistosoma mansoni* (trematode), and ES-62 (from nematode) have been found to modulate LPS induced TLR4 and TLR3 signaling pathway [11]. However, despite the progress in identifying some of the immunomodulatory helminth-derived molecules, the host receptors involved are largely unknown. Furthermore, immune suppressive mechanisms of some of the above mentioned factors require Th2 and IL-10 response to down regulate myeloid cell activation [21]. In this study we have focused on investigating the mechanism by which parasite antigens may block the initiation of the inflammatory pathway. This may represent a novel immune suppressive mechanism employed by helminths to prevent the development of the host inflammatory response to a variety of inflammatory stimuli. In this context, the physiological analysis in the present study suggests that helminth factors disrupt TRPC1-STIM1 interaction necessary for SOCE. The other possibility could be that parasitic ligands could directly inhibit TRPC1 function and target its degradation, which is currently under investigation. Our data also do not rule out an additional contribution of PL-PRR dependent modulation of second messenger response, leading to the down-regulation of the store depletion and inhibition of SOCE. However, as a similar inhibitory effect was observed of PL on Tg-induced store release, which does not involve any interaction with PRR. Furthermore, immediate addition of PL also inhibited TRPC1 activation and SOCE. Interestingly, it has been shown that *T. solium* and *M. corti* larvae have a tegument rich in glycan and poly basic antigens that, during CNS infection, are shed and taken up by host cells in the CNS environment [61]. Thus, we speculate that recognition of novel parasite antigens by Ca^2+^ channel proteins might be involved in this process. Currently we are testing putative parasite-derived antigens, including glycans, which interact with Ca^2+^ channel proteins and could have a role in the inhibition of agonist induced TRPC1-SOCE and NFκB pathway activation.

It has been previously demonstrated that in NCC patients, despite an overall inflammatory response observed in the CNS, macrophages around the metacestodes display a low expression of MHC-II molecules [61]. In contrast, MHC-II expression appears to be higher in cells located further away from the parasite [61]. Similar results were also found in studies involving viable cysts from *T. solium* infected pigs [62], [63]. Results from the present study demonstrate that, similar to human NCC, the murine model of NCC shows downregulated expression of MHC-II expression in many CD11b+ infiltrating myeloid cells in parasite infected brains. In addition, expression of costimulatory molecules was detected on fewer cells. Moreover, exposure to PL resulted in downregulation of multiple APC maturation markers *in vitro*. Indeed helminths have been shown to induce immune suppression, primarily due to the ability of helminth antigens to drive the immune bias toward a Th2 type response [12], [18], [64]. Th2 responses activate or expand AAMs, which express various negative signaling accessory molecules that induce T cell anergy and downregulate the proliferation of activated T cells [18], [65], [66]. However, the cytokines produced in the CNS during *M. corti* infection are indicative of a mixed T helper response but with IL-4, IL-13 and IL-10 detected in relatively low amounts [27]. Thus, the results here support suppression of innate immune pathways resulting in inhibition of myeloid cell maturation in NCC. In support, the secreted antigens of the cestodes have been shown to suppress mitogenic responses of human peripheral blood mononuclear cells [67] and depress proliferative response [68] and activation of CD4+ T cells [69]. Taken together, these observations may particularly highlight important mechanisms for macroscopic cestodes to persist in the host without inducing any detectable inflammatory response, including in NCC.

In NCC, it has been assumed that viable cysticerci induce immune suppressive effects and loss of these effects upon death of parasite likely leads to activation of PRRs resulting in uncontrolled inflammatory response and neuropathology [2], [6], [8]. Our previous studies using murine NCC model have identified that indeed TLR mediated response contributes to CNS inflammatory response and disease severity [29], [70]. The present study demonstrates that parasite soluble teguments antigens down-regulate the TLR ligation induced activation of BMDMs. We have now made the exciting observation that although, parasite antigens themselves do not modulate basal level of cytosolic Ca^2+^ turnover, they abolished the inflammatory stimuli e.g. LPS and Tg, -induced increase in intracellular Ca^2+^ accumulation and TRPC1-SOCE. As such Ca^2+^ entry into the cells is the first step of signaling processes that activates NFκB activation by inflammatory stimuli and plays central role in development/induction of host inflammatory response. Thus, inhibition of Ca^2+^ mediated inflammatory pathways likely is a novel helminth-induced immunosuppressive mechanism.
