# Correction: Helminth Induced Suppression of Macrophage Activation Is Correlated with Inhibition of Calcium Channel Activity

**DOI:** 10.1371/journal.pone.0111349

**Published:** 2014-10-13

**Authors:** The PLOS ONE Staff

There were some missing grant numbers in the funding statement. Please refer to the complete funding statement below:

This work was supported by grants RDC21418401001905 and seed grant awarded to BBM from NIH grant P30GM103329 (PI- Jonathan Geiger), NIH grants R01DE017102, and 1R03AI097532 awarded to BBS. The funders had no role in study design, data collection and analysis, decision to publish, or preparation of the manuscript.
